# miR-21 Reduces Hydrogen Peroxide-Induced Apoptosis in c-kit^+^ Cardiac Stem Cells In Vitro through PTEN/PI3K/Akt Signaling

**DOI:** 10.1155/2016/5389181

**Published:** 2016-10-10

**Authors:** Wenwen Deng, Yan Wang, Xianping Long, Ranzun Zhao, Zhenglong Wang, Zhijiang Liu, Song Cao, Bei Shi

**Affiliations:** ^1^Department of Cardiology, Affiliated Hospital of Zunyi Medical College, Zunyi 563000, China; ^2^Department of Anesthesiology, Zunyi Medical College, Zunyi 563000, China; ^3^Guizhou Key Laboratory of Anesthesia and Organ Protection, Zunyi Medical College, Zunyi 563000, China

## Abstract

The low survival rate of cardiac stem cells (CSCs) in the infarcted myocardium hampers cell therapy for ischemic cardiomyopathy. MicroRNA-21 (miR-21) and one of its target proteins, PTEN, contribute to the survival and proliferation of many cell types, but their prosurvival effects in c-kit^+^ CSC remain unclear. Thus, we hypothesized that miR-21 reduces hydrogen peroxide- (H_2_O_2_-) induced apoptosis in c-kit^+^ CSC and estimated the contribution of PTEN/PI3K/Akt signaling to this oxidative circumstance. miR-21 mimics efficiently reduced H_2_O_2_-induced apoptosis in c-kit^+^ CSC, as evidenced by the downregulation of the proapoptosis proteins caspase-3 and Bax and upregulation of the antiapoptotic Bcl-2. In addition, the gain of function of miR-21 in c-kit^+^ CSC downregulated the protein level of PTEN although its mRNA level changed slightly; in the meantime, miR-21 overexpression also increased phospho-Akt (p-Akt). The antiapoptotic effects of miR-21 were comparable with Phen (bpV), the selective inhibitor of PTEN, while miR-21 inhibitor or PI3K's inhibitor LY294002 efficiently attenuated the antiapoptotic effect of miR-21. Taken together, these results indicate that the anti-H_2_O_2_-induced apoptosis effect of miR-21 in c-kit^+^ CSC is contributed by PTEN/PI3K/Akt signaling. miR-21 could be a potential molecule to facilitate the c-kit^+^ CSC therapy in ischemic myocardium.

## 1. Introduction

Ischemic heart disease is still the leading cause of deaths worldwide. Despite advances in medicine, such as the catheter-based therapies, the 5-year mortality rate for myocardial infarction remains as high as 50% [1]. Alternative strategies, such as stem cell-based therapies, are urgently needed [2].

Stem cell-based therapies are promising in repairing cardiac damage due to ischemia-reperfusion (I/R) injury [3, 4]. Among various types of stem cells being investigated, c-kit^+^ cardiac stem cells (CSCs) appeared to be particularly promising because they are capable of differentiating into cardiomyocytes, smooth muscle cells, and endothelial cells [5]. In the past decade, studies demonstrated the abilities of human and rodent c-kit^+^ CSCs to promote cardiac regeneration and attenuate myocardial infarction (MI) induced heart dysfunction and remodeling in various animal models [6–13]. A recent report showed the beneficial effects of c-kit^+^ CSCs on ventricular remodeling and dysfunction sustained for more than one year in rats [7].

CSCs treatment of MI has demonstrated efficacy in the SCIPIO human Phase I trial [14]. After receiving CSCs, MI patients showed smaller infarct scars six months later. Despite the minimal cardiomyogenic potential of CSCs [7, 15], many researches have displayed their potential of promoting angiogenesis as well as decreasing apoptosis and necrosis in vivo, either via differentiation towards vascular lineages [16] or by secretion of growth factors [17] and/or extracellular microRNAs (miRNAs) [18].

However, poor engraftment and viability of CSCs minimize the percentage of cell survival and hamper functional improvements and cardiac outcomes [19]. The very poor survival of donor cells is one of the challenges that need to be overcome before CSC-based therapies become a clinical reality. It is reported that >90% of transplanted CSCs die within a week and >95% within 5 weeks in mice with MI [20, 21]. The massive loss of CSCs limits their effectiveness as a therapy. Strategies to enhance cell survival after adoptive transfer would produce notable therapeutic implications in post-MI patients. Strategies to increase cell survival include preconditioning the cells with a variety of techniques, including heat shock of the cells prior to transplantation, forced expression of survival factors in the donor cells, and exposure of cells to prosurvival factors [22–24]. Hu et al. improved the engraftment of transplanted CSCs and therapeutic efficacy for treatment of ischemic heart disease using a miRNA prosurvival cocktail, which contained miR-21, miR-24, and miR-221 [19].

miRNAs are small noncoding RNAs, which inhibit translation or promote mRNA degradation of their target genes [25, 26]. Accumulating evidence indicates that miR-21 plays important roles in tumor growth [27], vascular smooth muscle cell survival, and cardiac cell growth [28]. miRNAs also play critical roles in cardiogenesis and cardiac regeneration [29–32]. Gain-of-function studies indicated that miR-21 reduces cardiomyocyte apoptosis under oxidative stress [33, 34]. Importantly, the miRNA expression is capable of controlling CSCs fate and holds the potential of enhancing clinical efficacy of cellular therapy [19, 31]. It is reported that miRNAs also contribute to CSC differentiation [35–37]. For example, miR-21 not only modulates the immunoregulatory function of bone marrow mesenchymal stem cells (BMSCs) through the PTEN/Akt/TGF-*β*1 pathway [38], but also enhances human multipotent cardiovascular progenitors therapeutic effects via PTEN/HIF-1*α*/VEGF-A signaling [39].

The phosphatase and tensin homolog deleted on chromosome ten (PTEN), which was found as a tumor suppressor gene, participates in growth, apoptosis, adhesion, invasion, and migration [40, 41]. Silencing of PTEN can promote cell proliferation [42]. Pharmacological inhibition of PTEN limits myocardial infarct size and improves left ventricular function after MI [43]. PTEN works partially through the prosurvival pathway by inhibiting phosphorylation of Akt (p-Akt) which is the active form of Akt [41]. The overexpression of PTEN increases apoptosis in cardiomyocytes, whereas the inactivation of PTEN activates the Akt signaling, reduces apoptosis, and increases survival [44–47]. It is well documented that PTEN is one of miR-21's target genes [38, 48–50]. Accumulating evidence indicates that miR-21 promotes cell proliferation via PTEN-dependent PI3K/Akt activation in cancer cells [51–55]. Gain of function of miR-21 can efficiently reduce I/R injury by downregulation of the expression of PTEN [56, 57].

In this study, we provide evidence that miR-21 may protect c-kit^+^ CSCs against H_2_O_2_-induced apoptosis through the PI3K/PTEN/Akt signaling. This suggests that miR-21 possesses the ability to protect c-kit^+^ CSCs from oxidative injury and miR-21 could be a potential molecule to facilitate stem cell treatment of ischemic myocardium.

## 2. Materials and Methods

### 2.1. Animals

Sprague-Dawley rats (male, about 3 weeks old, 45–60 g) were purchased from the Third Military Medical University (Chongqing, China) and maintained in Zunyi Medical College. They were kept in 12 h light/dark (with 8:00 a.m. to 8:00 p.m. light on) cycles and were given free access to rat chow and water. All experimental procedures were performed according to the* Guide for the Care and Use of Laboratory Animals* in China and approved by the local Experimental Animal Care and Use Committee.

### 2.2. Materials

PE conjugated anti-CD34 and anti-CD45 primary antibodies were from BioLegend (USA). Collagenase type II was from Sigma (USA). Ham's/F-12 medium and fetal bovine serum (FBS) were purchased from HyClone (USA). Trypsin was purchased from Gibco (USA). Penicillin and streptomycin were from Solarbio (China). Fibroblast growth factor was from Peprotech (USA). Leukocyte inhibitory factor was a product of Gibco (USA). Rabbit anti-rat c-kit^+^ primary antibody was supplied by Biorbyt (UK). M-280 beads conjugated with sheep anti-rabbit secondary antibody were from Dynal Biotech (Norway). miR-21 mimics, miR-21 inhibitor, and their scrambles were synthesized by RIBOBIO (China). Lipofectamine 2000 was from Invitrogen (USA). Primers, miRNA reverse transcription kit, and qRT-PCR kit were from Sangon Biotech (China). Anti-*β*-actin, anti-Bcl-2, anti-Bax, anti-caspase-3, anti-PTEN, anti-p-Akt, and anti-Akt primary antibody and other secondary antibodies were obtained from Boster (China). Annexin V-FITC apoptosis detecting kit was from Solarbio (China). LY294002 (PI3K inhibitor) was from Beyotime Technology (China). Phen (bpV, PTEN inhibitor) was a product of Merck (Germany). The unlisted reagents were of analytical grade.

### 2.3. c-kit^+^ Cells Isolation, Purification, and Identification

CSCs were isolated [58] and purified [59] using previously published methods, with some modifications. Rats were deeply anesthetized with sevoflurane, and the atrial appendage was sliced and digested with 0.1% collagenase type II (Sigma, USA). After about 40 min digestion at 37°C, cells were collected by sedimentation at 1200 rpm for 5 minutes (min). Then, cells from the atrial appendage were incubated in a humidity chamber in Ham's F12 medium containing 10% FBS, 1% penicillin and streptomycin, 1% L-glutamine, 20 ng/mL human recombinant fibroblast growth factor, 20 ng/mL leukocyte inhibitory factor, and 10 ng/mL epidermal growth factor (EGF). When cells confluence reached >90%, they were suspended by trypsinization. Then, CSCs were incubated with rabbit anti-c-kit antibody (1 : 250 in F12 medium) for 1 hour (h) and sorted out with anti-rabbit secondary antibody conjugated 2.8 *μ*m magnetic beads (Dynal Biotech, Norway) in 30 min as instructed by the manufacturer's protocols. The purified c-kit^+^ CSCs were cultured in the aforementioned F12 medium. Flow cytometry was used to confirm the expression patterns of CSCs markers. Cells were incubated with fluorochrome-conjugated primary antibodies: anti-CD34-PE, anti-CD45-PE, and anti-c-kit primary antibody and anti-c-kit IgG-allophycocyanin (APC) secondary antibody (all from BioLegend, USA).

### 2.4. H_2_O_2_-Induced Oxidative Stress Model and Its Effect on Apoptosis in CSCs

Harvested CSCs were incubated in serum-free F12 medium for 24 h before being treated with H_2_O_2_ (0, 50, 100, and 200 *μ*M) for 2 h. Early apoptosis and necrosis of CSCs were determined by flow cytometry using Annexin V-FITC/PI staining assay as reported elsewhere [34]. The phosphatidylserine level on CSC surface was estimated with Annexin V-FITC and propidium iodide (PI) apoptosis detection kit (Solarbio, China) according to the manufacturer's instructions. Apoptosis and necrosis of c-kit^+^ CSCs were analyzed in a FACSCalibur flow cytometer (BD Biosciences, USA). Results were expressed as the percentage of apoptotic or necrotic cells from total cells. Flow cytometry was performed thrice using CSCs from three independent experiments.

### 2.5. Reverse Transcription and Real-Time PCR of miR-21 and PTEN

mRNA and miRNA levels were determined by using quantitative RT-PCR as previously reported [60, 61]. Briefly, RNAs from CSCs were isolated by the TRIzol (Invitrogen, USA) method. RT-PCR was performed on cDNA generated from 3 *μ*g of total RNA with a cDNA synthesis kit (TaKaRa, Japan) according to the manufacturer's protocol. RT-qPCR was performed with the CFX Connect Real-Time system (Bio-Rad, USA) using a SYBR Green PrimeScript RT kit (TaKaRa, Japan) based on the manufacturer's instructions. The PCR conditions included predenaturation at 95°C for 30 s followed by 40 cycles of denaturation at 95°C for 10 s and combined annealing/extension at 58°C for 30 s. All the mRNA expression levels were calculated based on the comparative quantification method (2^−ΔΔCT^). U6 and *β*-actin were used as internal controls for miR-21 and PTEN mRNA quantitation, respectively.

### 2.6. miR-21 Mimics and Inhibitor Transfection and the Detection of miR-21's Effects on Apoptosis in CSCs

For the miR-21 gain-of-function and loss-of-function experiments, miR-21 mimics, miR-21 inhibitor, and their control scrambles were added in 1.5 mL F12 medium in 6-well plates with 5 *μ*L transfection reagent Lipofectamine 2000 (Invitrogen, USA) and then incubated with c-kit^+^ CSCs for 48 h according to the manufacturer's instructions. Early apoptosis and necrosis of CSCs were determined by flow cytometry using Annexin V-FITC/PI staining assay as previously mentioned. Flow cytometry was performed twice using c-kit^+^ CSCs from three independent experiments.

### 2.7. Immunofluorescence of Caspase-3

To characterize purity of isolated CSCs, immunocytochemistry was used to verify c-kit expression on purified cells as reported elsewhere [62]. Cells were fixed with 4% paraformaldehyde and then blocked with 10% goat serum before being incubated with anti-caspase-3 antibody. c-kit^+^ CSCs were subsequently incubated with FITC-conjugated secondary antibody. After washing, the nuclei were counterstained with DAPI. The immunofluorescence photos were taken with a fluorescence microscope (Olympus, Japan).

### 2.8. Western Blot

Western blot analysis of total protein from c-kit^+^ cell lysis was performed as described previously [63]. The protein extracts were separated by SDS-polyacrylamide gels electrophoresis (SDS-PAGE) and transferred to PVDF membranes. After overnight blocking in nonfat milk solution, membranes were probed with anti-PTEN, anti-phospho-Akt, anti-Akt, anti-caspase-3, anti-Bax, anti-Bcl-2, anti-*β*-actin, or anti-GAPDH primary antibodies. PVDF membranes were incubated with horseradish peroxidase-conjugated secondary antibodies for 1 h and then enhanced chemiluminescence (Amersham Biosciences, USA). Immunoreactivity was visualized by a ChemiDoc MP system (Bio-Rad, USA). Protein levels were normalized to *β*-actin or GAPDH.

### 2.9. Statistical Analysis

Data are presented as mean ± SD. All data were analyzed by Student's *t*-test or by one-way ANOVA followed by LSD or Dunnett's T3 post hoc test for multiple comparisons. A *P* value of less than 0.05 was considered to be statistically significant. Data analyses were carried out using SPSS (v.19.0, IBM, USA).

## 3. Results

### 3.1. Isolated c-kit^+^ CSCs

**Figure 1:**
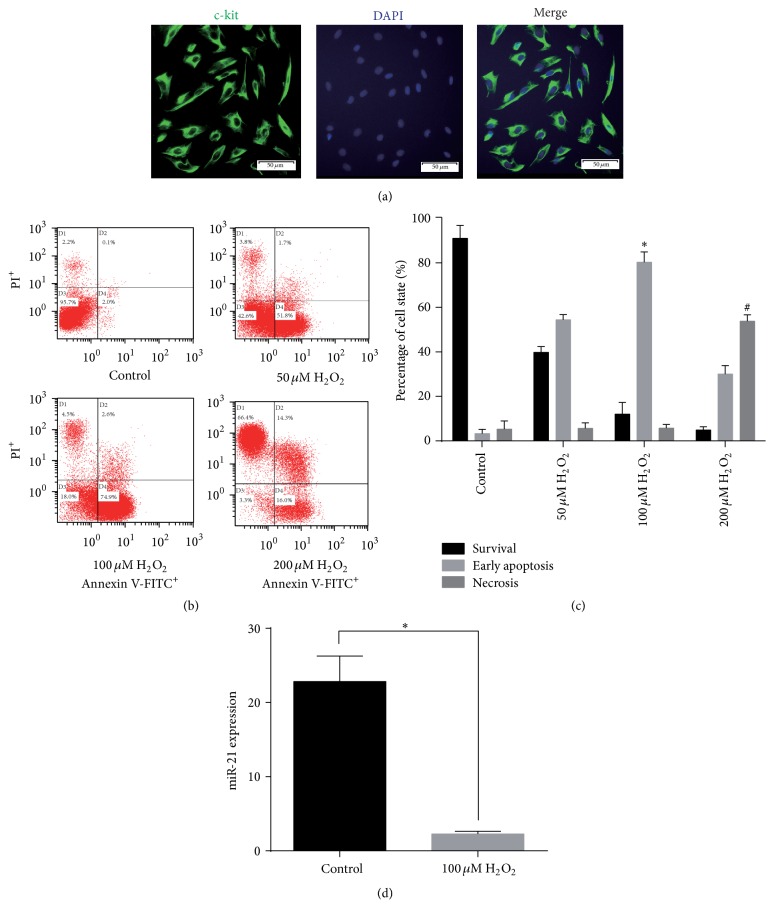
Effect of H_2_O_2_ (50, 100, and 200 *μ*M) on CSCs. After isolation from rat atrial appendage, cells were purified by combined use of c-kit antibody and magnetic beads conjugated with secondary antibody. Flow cytometry showed that c-kit^+^ cells were more than 90%. (a) Purified cells were double stained by c-kit (green) and DAPI (blue) and observed under a fluorescence microscope (Olympus, Japan). (b) The apoptosis rate of CSCs exposed to different concentrations of H_2_O_2_ for 2 h was measured using the Annexin V-FITC/PI staining assay and statistically calculated with flow cytometry. PI: propidium iodide. (c) The statistics of the states of CSCs in (b). ^*∗*^ *P* < 0.05 compared with the apoptosis rate in other groups; ^#^ *P* < 0.05 compared with the necrosis rate in other groups. *n* = 3. (d) H_2_O_2_'s effects on miR-21 expression. ^*∗*^ *P* < 0.05. *n* = 3 for both groups.

c-kit^+^ CSCs were isolated from rat atrial appendage and purified using anti-rabbit secondary antibody conjugated magnetic beads. Flow cytometry showed that 90.2% of cells were c-kit positive after the purification. Purified cells were stained with anti-c-kit antibody and counterstained with DAPI to visualize the nuclei. The immunofluorescence staining also showed a high percentage of double staining of c-kit^+^ and DAPI (Figure 1(a)).

### 3.2. H_2_O_2_ Induces Apoptosis and miR-21 Downregulation in CSCs

To establish an in vitro model of CSC apoptosis, H_2_O_2_ (50, 100, and 200 *μ*M) was selected to stimulate CSCs for 2 h. Flow cytometry results indicated that, after 2 h incubation, control showed no change, and 50 *μ*M and 100 *μ*M H_2_O_2_ induced 51.8% and 74.9% early apoptosis, respectively, compared with control (both *P* < 0.05). 100 *μ*M H_2_O_2_ challenge resulted in 74.9% apoptosis in CSCs (*P* < 0.05 compared with control or 100 *μ*M H_2_O_2_ group; Figures 1(b) and 1(c)), so we chose 100 *μ*M H_2_O_2_ to induce apoptosis in the subsequent experiments. Compared with control, 100 *μ*M H_2_O_2_ also induced the upregulation of proapoptotic proteins caspase-3 and Bax and the downregulation of antiapoptotic protein Bcl-2, as shown in Figures 3(a)–3(c). In addition, H_2_O_2_ significantly reduced miR-21 mRNA expression compared with control (*P* < 0.05, Figure 1(d)).

### 3.3. Transfection of CSCs with miR-21 Mimics or Antagomir-21 Changes miR-21 Expression

**Figure 2:**
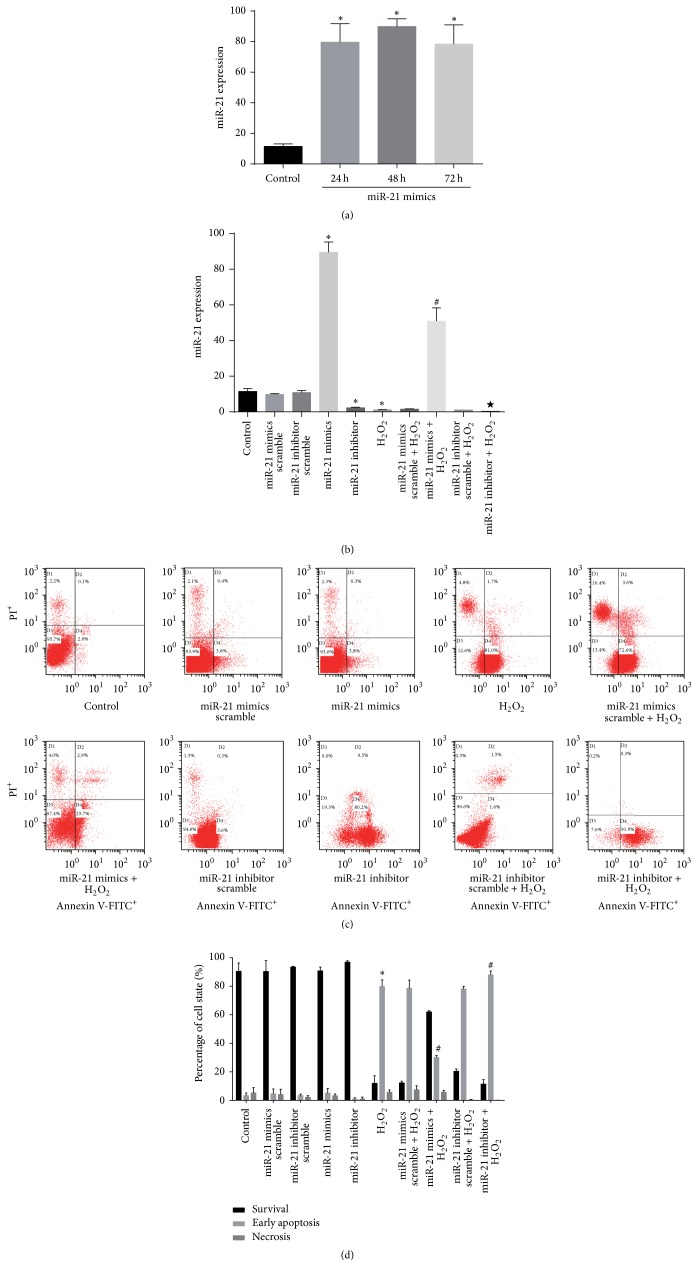
miR-21 mimics and inhibitor's effects on CSC apoptosis. (a) Cultured CSCs were treated with miR-21 mimics for 24, 48, or 72 h before miR-21 RT-PCR detection. miR-21 mimics significantly increased miR-21 but no difference was detected among the three time points. ^*∗*^ *P* < 0.05 compared with control. *n* = 3 in each group. (b) CSCs were incubated with miR-21 mimics, miR-21 inhibitor, or their scrambles for 48 h or challenged by 2 h incubation with 100 *μ*M H_2_O_2_ at the same time. miR-21 mimics significantly increased miR-21 levels, while miR-21 inhibitor significantly decreased miR-21 levels in normal or H_2_O_2_ stress condition. ^*∗*^ *P* < 0.05 compared with control; ^#^ *P* < 0.05 compared with H_2_O_2_ group; ^★^ *P* < 0.05 compared with miR-21 inhibitor group. *n* = 3 in each group. (c) The apoptosis rate of c-kit^+^ CSCs treated with mimics for 48 h and/or 100 *μ*M H_2_O_2_ for 2 h was measured using the Annexin V-FITC/PI staining assay. PI: propidium iodide. (d) The statistics of the states of CSCs in (c). ^*∗*^ *P* < 0.05 compared with control; ^#^ *P* < 0.05 compared with H_2_O_2_ group.

RT-PCR of miR-21 showed a significant increase of miR-21 when cells were transfected with miR-21 mimics 48 h later (*P* < 0.05 compared with control or miR-21 mimics scramble group; Figures 2(a) and 2(b)). The upregulation of miR-21 was stable in 72 h, and no difference was detected among 24, 48, and 72 h group (Figure 2(a)). We choose 48 h as the incubation time in the subsequent experiments. In addition, 48 h miR-21 inhibitor treatment decreased miR-21 expression compared with control or inhibitor scramble group (Figure 2(b)).

### 3.4. miR-21 Decreased H_2_O_2_-Induced Apoptosis in CSCs

**Figure 3:**
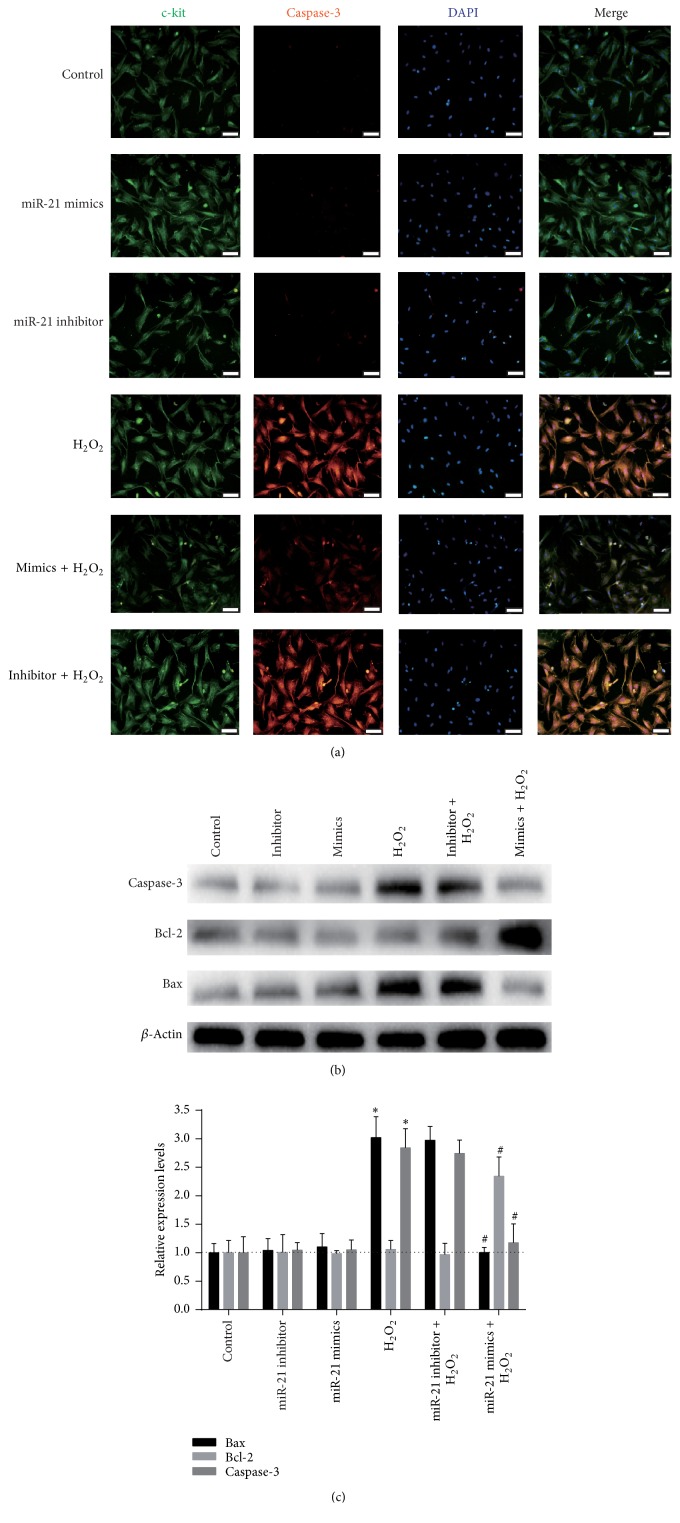
miR-21's effect on apoptosis related proteins in CSCs. Cultured CSCs were treated with miR-21 mimics, inhibitor, or scrambles for 48 h or challenged by 100 *μ*M H_2_O_2_ for 2 h at the same time. (a) c-kit^+^ CSCs were triple stained by c-kit (green), caspase (orange), and DAPI (blue) and observed under a fluorescence microscope (Olympus, Japan). Bar = 50 *μ*m. PI: propidium iodide. (b) miR-21 mimics' influences on Bax, Bcl-2, and caspase-3 detected with immune blotting. (c) Statistics of the relative expression level of proteins in (b). miR-21 mimics increased the expression of Bcl-2 and decreased Bax and caspase-3 compared with H_2_O_2_. ^*∗*^ *P* < 0.05 compared with control; ^#^ *P* < 0.05 compared with H_2_O_2_ group, *n* = 3.

The antiapoptotic effect of miR-21 was detected with flow cytometry using the Annexin V-FITC/PI staining assay. We found that miR-21 and its inhibitor have a little effect on apoptosis in normal cultured CSC, but miR-21 mimics significantly decreased CSC apoptosis after H_2_O_2_ insult (Figures 2(c) and 2(d)). Moreover, the caspase-3 and Bax expression was markedly decreased, while Bcl-2 apparently increased in the miR-21 mimics group, as determined by using immunofluorescence and western blot (Figure 3).

### 3.5. miR-21 Decreased PTEN Protein Expression

**Figure 4:**
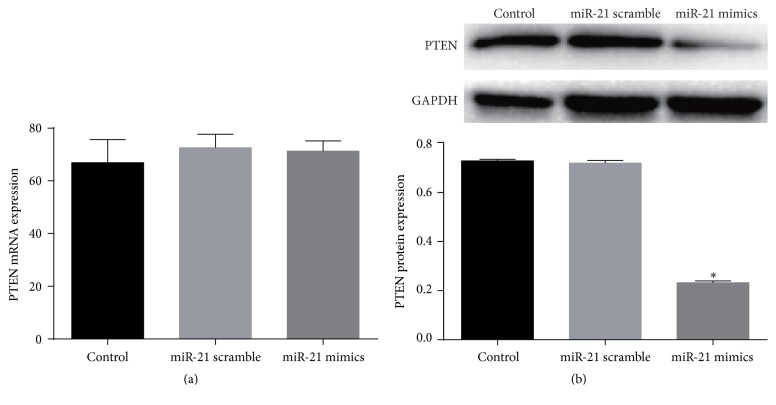
miR-21's effect on PTEN expression in CSCs under normal condition. Cultured CSCs were treated with miR-21 mimics or its negative control scramble for 48 h, and then cells were harvested and subjected to RT-PCR or western blot. PTEN mRNA of control, scramble treated, or miR-21 mimics treated cells showed no significant difference (a), but PTEN protein dramatically decreased after miR-21 mimics treatment (b). ^*∗*^ *P* < 0.05 compared with the other two groups. *n* = 3 in each group.

Although PTEN was extensively reported as one of miR-21's target genes in many cell types, western blot was employed to verify miR-mimic's effect on PTEN expression in c-kit^+^ CSCs. mRNA level of PTEN did not change (Figure 4(a)), while PTEN protein was significantly downregulated in mimics group as compared with control or miR-21 scramble group (*P* < 0.05, Figure 4(b)).

### 3.6. miR-21 Prevented CSCs from H_2_O_2_-Induced Apoptosis via the PTEN/PI3K/Akt Pathway

**Figure 5:**
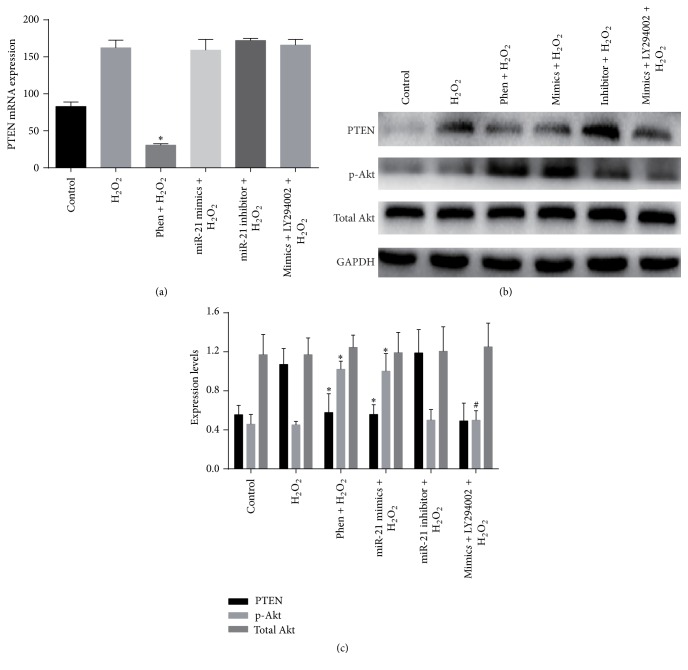
PTEN/PI3K/Akt pathway's contribution to miR-21 mimics' antiapoptotic effects. Cultured CSCs were treated with miR-21 mimics for 48 h before being subjected to 2 h H_2_O_2_ insult. PTEN and PI3K were inhibited with Phen or LY294002, respectively, before H_2_O_2_ insult. (a) RT-PCR was carried out to detect Phen, miR-21 mimics, and inhibitor's effects on PTEN expression. Phen significantly reduced the mRNA expression of PTEN. No change was detected among mimics + H_2_O_2_, inhibitor + H_2_O_2_, and H_2_O_2_ group. ^*∗*^ *P* < 0.05 compared with other groups. *n* = 3 in each group. (b-c) Western blot was carried out to detect Phen and miR-21 mimics' effects on PTEN and Akt protein expression. miR-21 mimics significantly downregulated PTEN protein in mimics + H_2_O_2_ group compared with H_2_O_2_ group. In addition, both Phen treatment and miR-21 mimics incubation increased p-Akt level, while PI3K inhibitor LY294002 decreased p-Akt level dramatically (*P* < 0.05). ^*∗*^ *P* < 0.05 compared with H_2_O_2_ group; ^#^ *P* < 0.05 compared with mimics + H_2_O_2_ group. *n* = 3 in each group. p-Akt: phospho-Akt.

**Figure 6:**
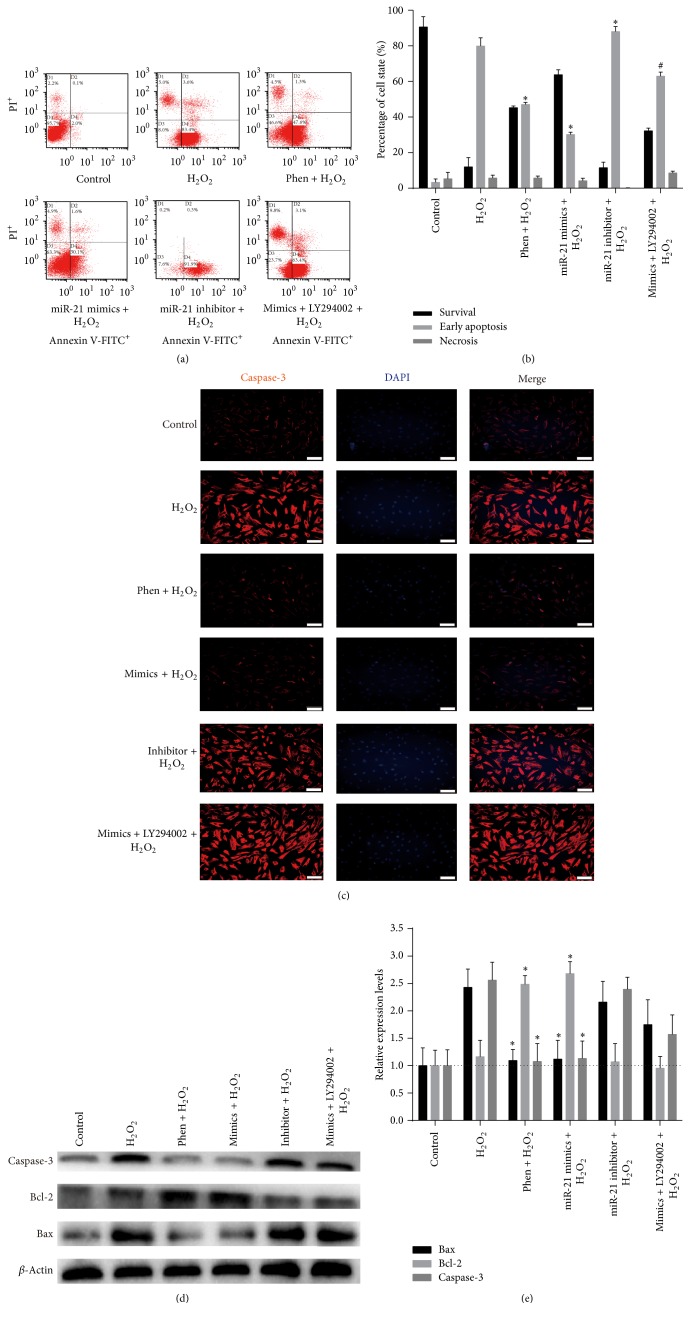
PTEN/PI3K/Akt pathway's contribution to H_2_O_2_-induced apoptosis in c-kit^+^ CSCs. Cultured CSCs were treated with miR-21 mimics or inhibitor for 48 h before being subjected to 2 h H_2_O_2_ insult. PTEN and PI3K were inhibited with Phen or LY294002, respectively, before H_2_O_2_ insult. (a) Flow cytometry was employed to detect necrosis and early apoptosis in CSCs that underwent different treatments. PI: propidium iodide. (b) Statistics of Annexin V-FITC/PI staining assay result in (a). When PTEN was inhibited by Phen, there was notably a decrease of necrosis and apoptosis in CSCs compared with H_2_O_2_ group. When PI3K was inhibited by LY294002, there was notably an increase of necrosis and apoptosis in mimics + LY294002 + H_2_O_2_ group compared with mimics + H_2_O_2_ group in CSCs. ^*∗*^ *P* < 0.05 compared with H_2_O_2_ group; ^#^ *P* < 0.05 compared with miR-21 mimics group. This trend was further confirmed by caspase-3 immunofluorescence tests (c) and caspase-3, Bax, and Bcl-2 immunoblotting tests (d-e). LY294002 reversed miR-21 mimics' effect on caspase-3, Bax, and Bcl-2, which is evidenced by the increase of caspase-3 (c–e) and Bax and the decrease of Bcl-2 (d-e). ^*∗*^ *P* < 0.05 compared with H_2_O_2_ group. *n* = 3 in each group. Bar = 50 *μ*m. PI: propidium iodide.

To study the mechanisms responsible for miR-21 mediated antiapoptotic effects in c-kit^+^ CSCs, we blocked PTEN and PI3K with their specific inhibitors Phen and LY294002, respectively. Molecular tests displayed that Phen significantly reduced the mRNA and protein expression of PTEN (Figures 5(a) and 5(b)). Both Phen and miR-21 mimics increased p-Akt level, while PI3K inhibitor LY294002 decreased p-Akt level dramatically (Figures 5(b) and 5(c)). Phen administration decreased apoptosis rate of CSCs under 100 *μ*M H_2_O_2_-induced stress just like the antiapoptotic effect of miR-21 mimics, while LY294002 partially reversed effects of miR-21 mimics (all *P* < 0.05, Figures 6(a) and 6(b)). LY294002 reversed miR-21 mimics' effect on caspase-3, Bax, and Bcl-2 expression, which was evidenced by the increase of caspase-3 and Bax and the decrease of Bcl-2 (Figures 6(c)–6(e)).

## 4. Discussion

Stem cell therapy is promising for the prevention and treatment of ischemic cardiomyopathy [1]. c-kit^+^ CSCs have emerged as one of the most potential CSCs [64]. Nevertheless, poor engraftment and viability limit the percentage of injected stem cells that contribute to cardiac functional improvements [19]. miRNAs hold the potential of improving engraftment and functional outcomes of cardiac progenitor cell transplantation [19, 31]. Studies have shown that miR-21 protects the myocardium against ischemic injury [61]. miR-21 also protects cardiomyocytes and BMSCs from H_2_O_2_-induced cell apoptosis and death. However, it is unknown whether miR-21 can influence CSCs survival under oxidative stress. Additionally, the underlying protective molecular mechanisms of miR-21 in CSCs need to be elucidated.

H_2_O_2_ has been widely used as an inducer of oxidative stress, which causes cell apoptosis [34]. In this study, we established an in vitro oxidative stress model with different concentration of H_2_O_2_ to simulate the microenvironment of infarcted myocardium. Given that 100 *μ*M H_2_O_2_ induced the highest apoptosis (up to 74.9%) and relatively low necrosis in c-kit^+^ CSCs, we chose 100 *μ*M H_2_O_2_ to study miR-21's prosurvival effects and mechanisms. We found that H_2_O_2_ induced the upregulation of proapoptosis proteins, caspase-3 and Bax, and the downregulation of antiapoptotic protein Bcl-2. Interestingly, our results showed that H_2_O_2_ reduced miR-21 expression in c-kit^+^ CSCs too. Although miR-21 was found to be downregulated by H_2_O_2_ treatment in H9C2 cell line [65] and BMSCs [27], most of the cardiac (myocytes, fibroblasts) and vascular cells (smooth muscle cells) treated with H_2_O_2_ showed increased miR-21 level [61, 66]. These results indicate that miR-21 expression patterns after H_2_O_2_ insult are cell specific. In addition, we should realize that miR-21 is a double-edged sword in ischemia-reperfusion injury, such as inflammation and angiogenesis [67]. This negative correlation between apoptosis and miR-21 expression indicates that miR-21 downregulation may aggravate apoptosis. This interaction was further confirmed by the gain-of-function analyses of miR-21, in which miR-21 significantly decreased c-kit^+^ CSC apoptosis, as well as the caspase-3 and Bax expression, and meanwhile increased Bcl-2 protein expression (Figures 3(a)–3(c)). These results indicate that miR-21 may be an antiapoptotic factor in c-kit^+^ CSCs under oxidative stress.

Although PTEN has been extensively reported as one of miR-21's target genes, it is not confirmed in CSCs to our knowledge. In many cell types, such as hepatocytes, cardiomyocytes, and cancer cells, miR-21 mediates the expression of PTEN [27, 49, 57]. We hypothesized that PTEN is a target gene of miR-21 in CSCs too. PTEN expression was directly calculated (Figure 3) after upregulation of miR-21 and the results confirmed our assumption. miR-21 upregulation caused significant downregulation of PTEN protein expression although the mRNA level of PTEN did not change much (Figures 3(a)–3(c)).

The PI3K/Akt pathway constitutes an approach to inhibit apoptosis and promote cell proliferation [68]. The activation of Akt significantly protects cells from H_2_O_2_-induced cell apoptosis [69, 70]. It has been reported that miR-21 affects the PI3K/Akt pathway through the expression of PTEN [49], but this effect has not been investigated in c-kit^+^ CSCs. To study whether the PTEN/PI3K/Akt signaling is responsible for miR-21 mediated antiapoptotic effect, we blocked PTEN and PI3K with their inhibitors Phen and LY294002, respectively, and then tested phosphorylation of Akt. Just like the prosurvival effects of miR-21, Phen administration decreased H_2_O_2_-induced apoptosis in c-kit^+^ CSCs (Figures 4(a) and 4(b)). For PI3K blocker LY294002, it partially reversed antiapoptotic effects of miR-21 mimics; at the same time, it attenuated miR-21's suppression of caspase-3 and Bax and the upregulation of Bcl-2 (Figures 6(c)–6(e)). Furthermore, both Phen and miR-21 mimics increased p-Akt level, while PI3K inhibitor LY294002 decreased p-Akt level drastically, which suggests that Akt is downstream of PI3K and PTEN. Although most literatures reported that Phen only downregulated the activity of PTEN but not its expression, some found that the mRNA and/or the protein expression was decreased after Phen administration [71, 72] as our results showed. We deem that the time window and the dose could be two influence factors. There may exist some feedback between PTEN activity and PTEN expression.

We found that the PTEN and PI3K inhibitors did not completely offset the prosurvival effects of miR-21. This is reasonable because miR-21 targets more than one gene, and, besides PTEN and PI3K mediated pathway, there could be other survival pathways involved. For example, miR-21 protects cardiac myocytes from the H_2_O_2_-induced injury by targeting PDCD4 gene, which is upstream of activator protein 1 (AP-1) [61]. AP-1 has been proved to be a key signaling molecule that determines life or death cell fates in response to extracellular stimuli including ROS. In addition, miR-21 promotes glioma invasion by targeting RECK and TIMP3 genes, which are suppressors of malignancy and inhibitors of matrix metalloproteinases [73]. PTEN also enhances human multipotent cardiovascular progenitors therapeutic effects via miR-21 initiated PTEN/HIF-1*α*/VEGF-A signaling. miR-21 in hESC-derived stage-specific embryonic antigen 1 (SSEA-1) positive cells inhibited phosphatase and tensin homolog (PTEN), which resulted in the activation of HIF-1*α* and the systemic release of VEGF-A [39].

In conclusion, our data reveal that miR-21 prevents CSCs from H_2_O_2_-induced apoptosis partially through the PTEN/PI3K/Akt pathway. The present study demonstrates that miR-21 is a prosurvival molecule for stressed c-kit^+^ CSCs. It also indicates that modification on miRNA expression may be able to enhance the clinical efficacy of cellular therapy.

We must confess some shortcomings of this study. In vivo studies are warranted to further confirm miR-21 and PTEN/PI3K/Akt pathway's effects on survival of c-kit^+^ CSCs. Besides, the luciferase assay could be a more direct way to confirm that PTEN is the target gene of miR-21.

## References

[1] Mozaffarian D., Benjamin E. J., Go A. S. (2016). Heart disease and stroke statistics—2016 update: a report from the American Heart Association. *Circulation*.

[2] Fisher S. A., Carolyn D., Anthony M., Enca M. R. (2015). Meta-analysis of cell therapy trials for patients with heart failure. *Circulation Research*.

[3] Hong K. U., Bolli R. (2014). Cardiac stem cell therapy for cardiac repair. *Current Treatment Options in Cardiovascular Medicine*.

[4] Sanganalmath S. K., Bolli R. (2013). Cell therapy for heart failure: a comprehensive overview of experimental and clinical studies, current challenges, and future directions. *Circulation Research*.

[5] Beltrami A. P., Barlucchi L., Torella D. (2003). Adult cardiac stem cells are multipotent and support myocardial regeneration. *Cell*.

[6] Bearzi C., Rota M., Hosoda T. (2007). Human cardiac stem cells. *Proceedings of the National Academy of Sciences of the United States of America*.

[7] Tang X.-L., Li Q., Rokosh G. (2016). Long-term outcome of administration of c-kit^POS^ cardiac progenitor cells after acute myocardial infarction: transplanted cells do not become cardiomyocytes, but structural and functional improvement and proliferation of endogenous cells persist for at least one year. *Circulation Research*.

[8] Bolli R., Tang X.-L., Sanganalmath S. K. (2013). Intracoronary delivery of autologous cardiac stem cells improves cardiac function in a porcine model of chronic ischemic cardiomyopathy. *Circulation*.

[9] Linke A., Müller P., Nurzynska D. (2005). Stem cells in the dog heart are self-renewing, clonogenic, and multipotent and regenerate infarcted myocardium, improving cardiac function. *Proceedings of the National Academy of Sciences of the United States of America*.

[10] Fischer K. M., Cottage C. T., Wu W. (2009). Enhancement of myocardial regeneration through genetic engineering of cardiac progenitor cells expressing Pim-1 kinase. *Circulation*.

[11] Angert D., Berretta R. M., Kubo H. (2011). Repair of the injured adult heart involves new myocytes potentially derived from resident cardiac stem cells. *Circulation Research*.

[12] Tang X.-L., Rokosh G., Sanganalmath S. K. (2010). Intracoronary administration of cardiac progenitor cells alleviates left ventricular dysfunction in rats with a 30-day-old infarction. *Circulation*.

[13] Taghavi S., Sharp T. E., Duran J. M. (2015). Autologous c-Kit+ mesenchymal stem cell injections provide superior therapeutic benefit as compared to c-Kit+ cardiac-derived stem cells in a feline model of isoproterenol-induced cardiomyopathy. *Clinical and Translational Science*.

[14] Bolli R., Chugh A. R., D'Amario D. (2011). Cardiac stem cells in patients with ischaemic cardiomyopathy (SCIPIO): initial results of a randomised phase 1 trial. *The Lancet*.

[15] van Berlo J. H., Kanisicak O., Maillet M. (2014). c-kit+ cells minimally contribute cardiomyocytes to the heart. *Nature*.

[16] Tallini Y. N., Kai S. G., Craven M. (2009). c-kit expression identifies cardiovascular precursors in the neonatal heart. *Proceedings of the National Academy of Sciences of the United States of America*.

[17] Huang C., Gu H., Yu Q., Manukyan M. C., Poynter J. A., Wang M. (2011). Sca-1+ cardiac stem cells mediate acute cardioprotection via paracrine factor SDF-1 following myocardial ischemia/reperfusion. *PLoS ONE*.

[18] Gray W. D., French K. M., Ghosh-Choudhary S. (2015). Identification of therapeutic covariant microRNA clusters in hypoxia-treated cardiac progenitor cell exosomes using systems biology. *Circulation Research*.

[19] Hu S., Huang M., Nguyen P. K. (2011). Novel microRNA prosurvival cocktail for improving engraftment and function of cardiac progenitor cell transplantation. *Circulation*.

[20] Hong K. U., Li Q.-H., Guo Y. (2013). A highly sensitive and accurate method to quantify absolute numbers of c-kit+ cardiac stem cells following transplantation in mice. *Basic Research in Cardiology*.

[21] Hong K. U., Guo Y., Li Q.-H. (2014). c-kit+ cardiac stem cells alleviate post-myocardial infarction left ventricular dysfunction despite poor engraftment and negligible retention in the recipient heart. *PLoS ONE*.

[22] Laflamme M. A., Chen K. Y., Naumova A. V. (2007). Cardiomyocytes derived from human embryonic stem cells in pro-survival factors enhance function of infarcted rat hearts. *Nature Biotechnology*.

[23] Haider H. K., Ashraf M. (2010). Preconditioning and stem cell survival. *Journal of Cardiovascular Translational Research*.

[24] Mohsin S., Khan M., Toko H. (2012). Human cardiac progenitor cells engineered with Pim-I kinase enhance myocardial repair. *Journal of the American College of Cardiology*.

[25] Small E. M., Frost R. J. A., Olson E. N. (2010). MicroRNAs add a new dimension to cardiovascular disease. *Circulation*.

[26] Bartel D. P. (2004). MicroRNAs: genomics, biogenesis, mechanism, and function. *Cell*.

[27] Lv C., Hao Y., Tu G. (2016). MicroRNA-21 promotes proliferation, invasion and suppresses apoptosis in human osteosarcoma line MG63 through PTEN/Akt pathway. *Tumor Biology*.

[28] Cheng Y., Zhang C. (2010). MicroRNA-21 in cardiovascular disease. *Journal of Cardiovascular Translational Research*.

[29] Anton R., Chatterjee S. S., Simundza J., Cowin P., DasGupta R. (2011). A systematic screen for micro-RNAs regulating the canonical Wnt pathway. *PLoS ONE*.

[30] Fuller A. M., Qian L. (2014). MiRiad roles for microRNAs in cardiac development and regeneration. *Cells*.

[31] Hosoda T. (2013). The mircrine mechanism controlling cardiac stem cell fate. *Frontiers in Genetics*.

[32] Thum T., Galuppo P., Wolf C. (2007). MicroRNAs in the human heart: a clue to fetal gene reprogramming in heart failure. *Circulation*.

[33] Wei C., Li L., Kim I. K., Sun P., Gupta S. (2014). NF-*κ*B mediated miR-21 regulation in cardiomyocytes apoptosis under oxidative stress. *Free Radical Research*.

[34] Lv C., Hao Y., Han Y. (2016). Role and mechanism of microRNA-21 in H_2_O_2_-induced apoptosis in bone marrow mesenchymal stem cells. *Journal of Clinical Neuroscience*.

[35] van Rooij E., Sutherland L. B., Qi X., Richardson J. A., Hill J., Olson E. N. (2007). Control of stress-dependent cardiac growth and gene expression by a microRNA. *Science*.

[36] Zhao Y., Samal E., Srivastava D. (2005). Serum response factor regulates a muscle-specific microRNA that targets Hand2 during cardiogenesis. *Nature*.

[37] Hosoda T., Zheng H., Cabral-Da-Silva M. (2011). Human cardiac stem cell differentiation is regulated by a mircrine mechanism. *Circulation*.

[38] Wu T., Liu Y., Fan Z. (2015). miR-21 modulates the immunoregulatory function of bone marrow mesenchymal stem cells through the PTEN/Akt/TGF-*β*1 pathway. *STEM CELLS*.

[39] Richart A., Loyer X., Néri T. (2014). MicroRNA-21 coordinates human multipotent cardiovascular progenitors therapeutic potential. *Stem Cells*.

[40] Ciuffreda L., Falcone I., Incani U. C. (2014). PTEN expression and function in adult cancer stem cells and prospects for therapeutic targeting. *Advances in Biological Regulation*.

[41] Panigrahi A. R., Pinder S. E., Chan S. Y., Paish E. C., Robertson J. F. R., Ellis I. O. (2004). The role of PTEN and its signalling pathways, including AKT, in breast cancer; an assessment of relationships with other prognostic factors and with outcome. *The Journal of Pathology*.

[42] Gregorian C., Nakashima J., Le Belle J. (2009). Pten deletion in adult neural stem/progenitor cells enhances constitutive neurogenesis. *The Journal of Neuroscience*.

[43] Keyes K. T., Xu J., Long B., Zhang C., Hu Z., Ye Y. (2010). Pharmacological inhibition of PTEN limits myocardial infarct size and improves left ventricular function postinfarction. *American Journal of Physiolog—Heart and Circulatory Physiology*.

[44] Mocanu M. M., Yellon D. M. (2007). PTEN, the Achilles' heel of myocardial ischaemia/reperfusion injury?. *British Journal of Pharmacology*.

[45] Schmid A. C., Byrne R. D., Vilar R., Woscholski R. (2004). Bisperoxovanadium compounds are potent PTEN inhibitors. *FEBS Letters*.

[46] Schwartzbauer G., Robbins J. (2001). The tumor suppressor gene *PTEN* can regulate cardiac hypertrophy and survival. *The Journal of Biological Chemistry*.

[47] Wu D.-N., Pei D.-S., Wang Q., Zhang G.-Y. (2006). Down-regulation of PTEN by sodium orthovanadate inhibits ASK1 activation via PI3-K/Akt during cerebral ischemia in rat hippocampus. *Neuroscience Letters*.

[48] Stambolic V., Suzuki A., De la Pompa J. L. (1998). Negative regulation of PKB/Akt-dependent cell survival by the tumor suppressor PTEN. *Cell*.

[49] Qi W., Li H., Cai X.-H. (2015). Lipoxin A4 activates alveolar epithelial sodium channel gamma via the microRNA-21/PTEN/AKT pathway in lipopolysaccharide-induced inflammatory lung injury. *Laboratory Investigation*.

[50] Li J., Yen C., Liaw D. (1997). PTEN, a putative protein tyrosine phosphatase gene mutated in human brain, breast, and prostate cancer. *Science*.

[51] Meng F., Henson R., Lang M. (2006). Involvement of human micro-RNA in growth and response to chemotherapy in human cholangiocarcinoma cell lines. *Gastroenterology*.

[52] Di Cristofano A., Pandolfi P. P. (2000). The multiple roles of PTEN in tumor suppression. *Cell*.

[53] Bai H., Xu R., Cao Z., Wei D., Wang C. (2011). Involvement of miR-21 in resistance to daunorubicin by regulating PTEN expression in the leukaemia K562 cell line. *FEBS Letters*.

[54] Yan-Nan B., Zhao-Yan Y., Li-Xi L., Qing-Jie X., Yong Z. (2014). MicroRNA-21 accelerates hepatocyte proliferation in vitro via PI3K/Akt signaling by targeting PTEN. *Biochemical and Biophysical Research Communications*.

[55] Ou H., Li Y., Kang M. (2014). Activation of miR-21 by STAT3 induces proliferation and suppresses apoptosis in nasopharyngeal carcinoma by targeting PTEN gene. *PLoS ONE*.

[56] Sayed D., He M., Hong C. (2010). MicroRNA-21 is a downstream effector of AKT that mediates its antiapoptotic effects via suppression of fas ligand. *The Journal of Biological Chemistry*.

[57] Tu Y., Wan L., Fan Y. (2013). Ischemic postconditioning-mediated miRNA-21 protects against cardiac ischemia/reperfusion injury via PTEN/Akt pathway. *PLoS ONE*.

[58] Choi S. H., Jung S. Y., Suh W., Baek S. H., Kwon S.-M. (2013). Establishment of isolation and expansion protocols for human cardiac C-kit-positive progenitor cells for stem cell therapy. *Transplantation Proceedings*.

[59] Kazakov A., Meier T., Werner C. (2015). C-kit+ resident cardiac stem cells improve left ventricular fibrosis in pressure overload. *Stem Cell Research*.

[60] Cao S., Liu Y., Sun W. (2015). Genome-wide expression profiling of anoxia/reoxygenation in rat cardiomyocytes uncovers the role of mito K_ATP_ in energy homeostasis. *Oxidative Medicine and Cellular Longevity*.

[61] Cheng Y., Liu X., Zhang S., Lin Y., Yang J., Zhang C. (2009). MicroRNA-21 protects against the H_2_O_2_-induced injury on cardiac myocytes via its target gene PDCD4. *Journal of Molecular & Cellular Cardiology*.

[62] Di Bernardini E., Campagnolo P., Margariti A. (2014). Endothelial Lineage differentiation from induced pluripotent stem cells is regulated by microRNA-21 and transforming growth factor *β*2 (TGF-*β*2) pathways. *The Journal of Biological Chemistry*.

[63] Cao S., Liu Y., Wang H. (2016). Ischemic postconditioning influences electron transport chain protein turnover in Langendorff-perfused rat hearts. *PeerJ*.

[64] Nigro P., Perrucci G. L., Gowran A., Zanobini M., Capogrossi M. C., Pompilio G. (2015). c-kit^+^ cells: the tell-tale heart of cardiac regeneration?. *Cellular & Molecular Life Sciences*.

[65] Xiao J., Pan Y., Li X. H. (2016). Cardiac progenitor cell-derived exosomes prevent cardiomyocytes apoptosis through exosomal miR-21 by targeting PDCD4. *Cell Death & Disease*.

[66] Lin Y., Liu X., Cheng Y., Yang J., Huo Y., Zhang C. (2009). Involvement of MicroRNAs in hydrogen peroxide-mediated gene regulation and cellular injury response in vascular smooth muscle cells. *The Journal of Biological Chemistry*.

[67] Xu X., Kriegel A. J., Jiao X. (2014). miR-21 in ischemia/reperfusion injury: a double-edged sword?. *Physiological Genomics*.

[68] Sussman M. (2007). ‘AKT’ing lessons for stem cells: regulation of cardiac myocyte and progenitor cell proliferation. *Trends in Cardiovascular Medicine*.

[69] Yang P., Peairs J. J., Tano R., Jaffe G. J. (2006). Oxidant-mediated Akt activation in human RPE cells. *Investigative Ophthalmology & Visual Science*.

[70] Byeon S. H., Lee S. C., Choi S. H. (2010). Vascular endothelial growth factor as an autocrine survival factor for retinal pigment epithelial cells under oxidative stress via the VEGF-R2/PI3K/Akt. *Investigative Ophthalmology & Visual Science*.

[71] Mao L.-L., Hao D.-L., Mao X.-W. (2015). Neuroprotective effects of bisperoxovanadium on cerebral ischemia by inflammation inhibition. *Neuroscience Letters*.

[72] Wang D.-F., Yang H.-J., Gu J.-Q. (2015). Suppression of phosphatase and tensin homolog protects insulin-resistant cells from apoptosis. *Molecular Medicine Reports*.

[73] Gabriely G., Wurdinger T., Kesari S. (2008). MicroRNA 21 promotes glioma invasion by targeting matrix metalloproteinase regulators. *Molecular & Cellular Biology*.

